# Supplementary material for: Preterm Infants on Early Solid Foods and Vitamin D Status in the First Year of Life—A Secondary Outcome Analysis of a Randomized Controlled Trial
Source: Nutrients. 2022 Jul 28;14(15):3105. doi: 10.3390/nu14153105 (PMC9370713; doi:10.3390/nu14153105)
Supplement: Supplementary file 1 [file nutrients-14-03105-s001.zip › nutrients-1804435-supplementary.pdf]

Table S1. Detailed results from the linear mixed effects model for serum vitamin D at all study visits.

Covariates	6 weeks corrected age		6 months corrected age		12 months corrected age	
	**estimate (95% CI)**	***p***	**estimate (95% CI)**	***p***	**estimate (95% CI)**	***p***
Group (late)	2.05 (−5.46 to 9.51)	0.59	3.51 (−0.35 to 7.37)	0.07	3.37 (−0.32 to 7.06)	0.07
Cumulative vitamin D supplementation (IE/days)	−0.03 (−0.07 to 0.01)	0.16	−0.01 (−0.03 to 0.01)	0.28	0.002 (−0.01 to 0.01)	0.76
Sex (male)	−6.30 (−13.04 to 0.44)	0.07	2.09 (−1.48 to 5.66)	0.23	1.99 (−1.11 to 5.08)	0.20
Gestational age at birth (days)	0.07 (−0.20 to 0.34)	0.58	−0.10 (−0.24 to 0.04)	0.15	−0.06 (−0.19 to 0.08)	0.38
Nutrition at discharge (formula)	−13.43 (−22.65 to -4.20)	0.005	−3.50 (−8.38 to 1.39)	0.16	−1.51 (−6.24 to 3.22)	0.53
Nutrition at discharge (mix)	−5.61 (−14.76 to 3.53)	0.23	−0.49 (−5.32 to 4.35)	0.84	−0.43 (−4.84 to 3.98)	0.85

For the evaluation of differences between the early and late feeding group the following fixed effects were included: cumulative vitamin D supplementation (IE/days), gestational age at birth, sex, nutrition at discharge. A random intercept was fit to account for possible correlation between siblings of multiple births. CI, Confidence interval.
